# Pro-Inflammatory Cytokines Reduce the Proliferation of NG2 Cells and Increase Shedding of NG2 *In Vivo* and *In Vitro*

**DOI:** 10.1371/journal.pone.0109387

**Published:** 2014-10-06

**Authors:** Malin Wennström, Shorena Janelidze, Cecilie Bay-Richter, Lennart Minthon, Lena Brundin

**Affiliations:** 1 Lund University, Department of Clinical Sciences, Clinical Memory Research Unit, Wallenberg Laboratory, Malmö, Sweden; 2 Aarhus University, Department of Clinical Medicine, Translational Neuropsychiatry Unit, Risskov, Denmark; 3 Michigan State University, College of Human Medicine, Department of Psychiatry and Behavioral Medicine, Grand Rapids, Michigan, United States of America; 4 Van Andel Research Institute, Laboratory of Behavioral Medicine, Grand Rapids, Michigan, United States of America; Universidad de Sevilla, Spain

## Abstract

Neuron glial 2 (NG2) cells become strongly activated in injured brain areas. The activation is characterized by increased proliferation as well as increased expression and shedding of the proteoglycan NG2 expressed on their cell surface. It is currently not known how these cells respond to low-grade neuroinflammation provoked by systemic inflammation. To investigate this, we analyzed NG2 cell proliferation as well as soluble NG2 (sNG2) in cerebrospinal fluid (CSF) from rats treated with an acute intraperitoneal (i.p) injection of lipopolysaccharides (LPS) or saline and sacrificed after 2 or 24 hours. The systemically induced neuroinflammation was confirmed as elevated levels of cytokines, including interleukin (IL)-6 and IL-1β, and MHCII expressing microglia were found 24 h after LPS treatment. At this time point NG2 cell proliferation was significantly decreased in both amygdala and hippocampus and sNG2 levels in CSF were increased twofold. We also exposed human NG2 cells in culture to IL-6 and IL-1β for 24 h and found, in line with our *in vivo* study, a direct impact of these cytokines reducing cell proliferation and increasing shedding of NG2. We conclude that LPS induced systemic inflammation significantly affects NG2 cell proliferation and shedding and that these two events at least in in part are mediated by IL-6 and IL-1β.

## Introduction

The neuron glial 2 (NG2) cell is a glial cell type representing the majority of proliferating cells in the central nervous system (CNS)[1], [2], [3], [4]. The cell population is primarily known to serve as an oligodendrocyte progenitor pool, but many of the NG2 cells, particularly in the gray matter, remain undifferentiated for a significant time. As such, they become well integrated into the cellular network, displaying several interesting properties including forming direct synapses with neurons (for review see[5], [6]). Although an increasing number of studies have started to chart the function and properties of NG2 cells, little is known about their role in pathological conditions. Interestingly, rodent studies have shown robust changes in the NG2 cell population in brain areas subjected to focal injury. After stab wound injury or kainite induced lesions, NG2 cells become activated, a state characterized by altered morphology, enhanced proliferation and increased expression of its cell characteristic surface bound proteoglycan NG2 [7], [8]. Also stereotactic microinjection of cytokines and lipopolysaccharide (LPS) into the rat brain robustly activates NG2 cells [9]. In addition, damage and neuroinflammatory processes affect the metabolism of surface bound NG2 proteoglycan, as studies have demonstrated an increased amount of soluble (i.e shedded) proteoglycan NG2 (sNG2) in stab-wounded rat cortex [10] and experimental autoimmune encephalomyelitis lesions[11].

The studies mentioned above provide evidence that severe focal injury greatly affects the NG2 cell population, which is of interest when investigating clinical conditions linked to direct brain damage such as stroke, wounds or concussions. However, milder or chronic alterations in the cellular environment may also provoke pathological conditions in the brain. Although the brain long was considered to be an immuno-privileged site [12] it has become clear that systemic inflammation can induce specific immunological mechanisms within the brain. The underlying mechanisms are not completely understood, but both direct transmission of peripherally derived cytokines across the blood-brain-barrier (BBB) and indirect effects on afferent nerves, such as the vagus nerve, have been described (for review see [13]). Studies using LPS to induce systemic inflammation show a marked increase of microglial activity. These alterations include enhanced MHC II expression [14], up-regulation of ED-1 (indicative of lysosomal activity) [15], morphological changes [14], [16], [17] and increased levels of the microglial derived proinflammatory cytokines, such as tumor necrosis factor alpha (TNF- α) [18] and interleukin 1β (IL-1β)[19]. Furthermore, post-mortem studies on brain tissue from patients with sepsis show increased microglia expression of ED-1 and MHC II [20] and patients with invasive meningococcal disease (IMD) are known to display elevated levels of several cytokines in the cerebrospinal fluid (CSF)[21].

Although there are several studies demonstrating microglial activity in response to systemic inflammation, there are to our knowledge few (if any) reports describing how the NG2 cell population reacts upon these pathological events. In the current study we therefore investigate whether systemically induced neuroinflammation, induced by intraperitoneal injection of LPS, affects the NG2 cell population in regard to proliferation and morphological changes. We also analyzed the sNG2 levels in CSF of the LPS treated rats to establish whether changes in sNG2 levels correlate to NG2 cell population alterations in the brain. Finally, in order to determine whether cytokines have a direct impact on proliferation of NG2 cells and sNG2 shedding, we stimulated human NG2-expressing oligodendrocyte progenitor cells (further referred to as human NG2 cells in this text) with the cytokines IL-8, IL-6, IL-4 and IL-1β.

## Materials and Methods

### Animals

Adult male Wistar rats (Harlan Laboratories, Holland) weighing 250–300 g were used. Experimental procedures were approved by the Swedish Board of Agriculture (Jordbruksverket) and carried out according to the guidelines set by the Ethical Committee for Use of Laboratory Animals in the Lund/Malmö region. Three rats were housed in each cage on a 12 hours light/dark cycle with access to food and water ad libitum. Since previous studies have shown that microglia and NG2 cells become reactive within 8–24 h after injury [8], [22] we conducted two studies with different survival time points, before and during glial activation i) LPS2h: 2 h survival after LPS (n  =  9) and saline treatment (n = 9) and ii) LPS24h: 24 h survival after LPS (n  =  8) and saline treatment (n = 9).

### Administration of lipopolysaccaride

Rats received a single intraperitoneal injection of 5 mg/kg of bacterial LPS (Escherichia coli serotype 055:B5; SIGMA, St Louis, MO) in sterile physiological saline. This dose is adequate to induce inflammation in the brain, but not lethal sepsis [18]. Control groups received sterile physiological saline.

### Sample collection

Rats were anesthetized with sodium pentobarbital 60 mg/ml and after the disappearance of nociceptive reflexes, the cerebrospinal fluid from the cisterna magna was collected according to procedure described previously [23]. Briefly, a stereotaxic instrument horizontally fixed the rat head, whereas the rest of the body was lying vertically. A needle connected to a syringe was inserted vertically between the occipital protuberance and the spine of the atlas and the CSF was collected with a gentle aspiration.

Blood samples were collected by cardiac puncture and kept at room temperature (RT) for 1 h, followed by centrifugation at 4°C, 1300 × g for 10 minutes and serum collection. CSF and serum samples were stored in −80°C. Finally, after the CSF and blood collection, the rats were transcardially perfused with 0.9% saline (50 ml), followed by 4% ice-cold paraformaldehyde (250 ml). Brains were postfixed in 4% paraformaldehyde at 4°C for 4 hours and thereafter in 30% sucrose in PBS until they sank. Coronal sections (40 µm) of the entire brain were cut on a freezing microtome and stored in antifreeze cryoprotectant solution at −20°C.

### Cytokine analysis

Multiplex ultra-sensitive electrochemiluminescence immunoassay (Meso Scale Discovery, UK) was used for quantification of the following cytokines in rat CSF and blood samples: interferon gamma (IFN- γ), interleukine-13 (IL-13), interleukin-1 beta (IL-1ß), interleukin-4 (IL-4), interleukin-5 (IL-5), interleukin-6 (IL-6), tumor necrosis factor alpha (TNF- α) and chemokine growth-related oncogeneCXCL1 (rGro/KC), as previously described [24]. Standards and samples were analyzed in duplicates. Detection limits for the different cytokines in serum were as follows: IFN- γ: 9.65 pg/ml, IL-13: 17 pg/ml, IL-1ß: 26.8 pg/ml, IL-4: 12.3 pg/ml, IL-5: 27.5 pg/ml,rGro/KC:132 pg/ml and TNF- α: 23.1 pg/ml. The detections limits for cytokines in CSF were: IFN- γ: 33.2 pg/ml, IL-13: 146 pg/ml, IL-1ß: 34.8 pg/ml, IL-4: 9.86 pg/ml, IL-5: 49.2 pg/ml, TNF- α: 131 pg/ml and rGro/KC:645 pg/ml. The detection limits for IL-6 in serum and CSF were17.7 and 35.2 pg/ml, respectively. Samples with zero cytokine levels were assigned the value of the respective detection limit.

### Immunohistological staining procedures

Reactive microglia and proliferating NG2 cells were investigated by double immunofluorescence stainings. The following primary antibodies were used: rabbit anti-NG2 (1:500, Millipore, Temecula, USA), rabbit anti-Iba-1 (1:200; Wako, Neuss, Germany) mouse-anti- RT1B against MHC II (1:500, AbDSerotec, Oxford, UK) and rabbit anti Ki67 (1:200, Abcam, Cambridge, UK). The latter antibody is directed against the endogenous proliferation marker Ki67 [25] and stain cells dividing at the time point of perfusion, which is of importance when assessing alteration in ongoing cell proliferation at different time points [26]. Sections were incubated with the primary antibodies (Iba-1/MHCII and NG2/ki67) overnight at 4°C and were the following day incubated with Alexa 488 conjugated goat anti-rabbit (1:200; Vector BA-1000, Vector Laboratories Inc., Burlingame, CA, USA) and Cy3 conjugated horse anti-mouse (1:200; Jackson Immunoresearch, West Grove, PA, USA) for 2 hours in room temperature (RT). Thereafter the sections were mounted in Vectashield anti-fading mounting medium with DAPI (Vector Laboratories, Burlingame, CA).

Alterations in NG2 immunoreactivity were investigated using immunohistochemistry (IHC).

### Data analysis of immunohistological stainings

Number of proliferating NG2-cells (NG2+/Ki67+) and MHCII expressing microglia were analyzed in the medial prefrontal cortex (mPFC), the basolateral nucleus (BL) of amygdala and the granular cell layer (GCL), the dentate hilus and the molecular layer (ML) of hippocampus. Analysis of the number of Ki67+ cells in the GCL was confined to the subgranular zone (SGZ) and cells extending into the dentate hilus, but being part of clusters of Ki67+ cells in the SGZ, were included. Cells were counted bilaterally through the mid-dorsal part of hippocampus (−2.8 mm to −4.52 mm relative to the bregma, 5 sections per animal). Amygdala was analyzed, according to previously described guidelines [27], [28], through the mid-part of the rostrocaudal extension of the basolateral nucleus (−2.65 mm to −3.40 mm relative to the bregma, 3 section per animal). Medial prefrontal cortex (−2.65 mm to −3.40 mm relative to the bregma, 3 section per animal) was analyzed according to previously described guidelines [29]. The chosen coordinates give a maximal cross-sectional diameter of the analyzed regions and a minimum change in counted area size per section. To further ensure that the sizes of the analyzed regions did not differ between treatment groups, we quantified the cross-sectional areas of the NG2-stained sections using CellSense dimension software (Olympus Optical Co., Ltd., Tokyo, Japan). None of the regions analyzed differed in size between treatment groups (Table S1). All Iba-1+, MHCII+ and NG2+/Ki67+ cells were counted using an Olympus BX41 epifluorescence microscope with a 40× objective (Olympus Optical Co., Ltd., Tokyo, Japan). When counting MHCII +cells, we differentiated between dendritic MHCII, indicative of activated microglia, and non-dendritic MHCII+ cells. Since NG2 is expressed on both pericytes and glial cells in the rat brain, we only counted NG2+ cells with the specific morphological features of NG2 expressing glial cells (i.e. dendritic NG2 cells). Before counting double-labeled cells in the epifluorescencemicroscope, we confirmed that our judgment of a double-labeled cell was correct using a confocal laser-scanning microscope (Leica TCS SL, Heidelberg, Germany). Number of cells per section and region was determined and the values were averaged and presented as the mean cell number per section and region.

### Cell culture studies

Primary fetal human brain oligodendrocyte progenitors bought from the commercial company ScienCell Research Laboratories (Carlsbad, USA) were cultured in OPC medium (SciencCell Research Laboratories, Carlsbad, USA). The cells were grown as monolayers in poly-L-lysine (PLL) coated culture flasks in humified air with 5% CO_2_ at 37°C until 80–90 % confluent. The cells were thereafter harvested by gentle scraping and plated in poly-L-lysine coated 96 well ViewPlates (Perkin Elmer, Waltham, MA USA) and grown until 70% confluent. For cell experiment the culture media was removed and replaced by OPC medium containing either 0.3 ng/ml human recombinant IL-1β, 150 pg/ml human recombinant IL-4, 1.5 ng/ml human recombinant IL-6 or 20 ng/ml human recombinant IL-8. The cytokine concentration chosen were based on the concentrations found in the rat CSF 2 h after LPS treatment. Cells exposed to normal OPC medium were used as controls cells. Labeling of proliferating cells was performed using Click-iT EdU Alexa Fluor 488 Imaging Kit (Life technologies, Waltham, MA USA) according to the suppliers instructions. The thymidine analog EdU was added to the cell cultures directly after cytokine supplementation. The cells were thereafter incubated for 24 h at 37°C in 5% CO_2_. The experiment was independently repeated 6 times in duplicates. After treatment the cell culture supernatants were collected, centrifuged (275× g, 5 min, 4°C), aliquoted and stored at −80°C until used. Cell culture purity was analyzed by immunocytoflourenscence staining against NG2 and PDGFR-AA. Cells were fixed with 2% formaldehyde and unspecific binding was blocked by PBS containing 1% BSA (Boehringer Mannheim, Kircheim, Germany) and 5% goat serum (Jackson immunoresearch, West Grove, USA). The cells were thereafter incubated with either mouse anti-human NG2 (1:200 Millipore, Darmstadt, Germany) or rabbit anti-PDGF-AAR in blocking solution (1:200 Abcam, Cambridge, UK followed by an incubation with the secondary antibodies (dylight549-conjugated anti-rabbit Igs (1:500 Molecular probes) or Cy3-conjugated anti-mouse Igs (1:500, Jackson Immunoresearch, West Grove, USA). Analysis of DAPI staining (n = 200) showed that the majority of DAPI positive cells (>97%) expressed NG2 and PDGFR-AA. Proliferation of cells was measured by analyzing the ratio of EdU positive cell area to DAPI positive cell area. The number of DAPI positive cells for each assessment was more than 200. The area of EdU positive cells and DAPI positive cells was determined using CellSense dimension software (Olympus Optical Co., Ltd., Tokyo, Japan).

### NG2 quantification assay

Levels of soluble NG2 in rat CSF and in supernatants from cultured human NG2 cells were analyzed with an in-house assay using the Mesoscale Discovery (MSD) electrochemiluminescence (ECL) technology employing immunoassay conversion kits (MSD, Rockville, USA) as described previously[30]. In brief, samples were diluted in PBS and coated onto MSD multi array plates in duplicate wells (25µl) and allowed to adhere overnight at 4°C. The following day wells were rinsed and incubated with blocking solution (1% BSA containing 1% milk in PBS-T) for 1 h at RT followed by another rinse and incubation with mouse anti NG2 antibody (1:100, clone B5, ATCC kind gift from Dr William Stallcup) for 2 h at RT on an orbital shaker. Wells were then rinsed and incubated with sulfo-Tag goat-anti mouse antibody (MSD, Rockville, USA) for 1 h in RT on an orbital shaker. Recombinant rat NG2 (kind gift from Dr William Stallcup) or recombinant human NG2 (Origene technologies) was used as standard. Resulting ECL signal was quantified using an MSD SECTOR Imager 6000. Readings of the duplicate standards and samples were averaged and NG2 concentrations determined by interpolation of a 4 parametric curve fit. Detection limit of the rat NG2 analysis was determined to be 33.40ng/ml, whereas detection limit of the human NG2 analysis was 2.28 pg/ml.

### Statistical analysis

Statistical analysis was performed using the SPSS software (version 20.0 for Windows, SPSS Inc., Chicago, IL, USA). Cytokine data was analyzed with Mann-Whitney Test due to a non normal distribution. The *in vivo* cell counting studies as well as the NG2 fraction study and fibrinogen area study were analyzed with student t-test. Differences in rat CSF NG2 concentrations were assessed by Student t-test. Data are presented as mean ± SEM with a statistical significance set to *p*<0.05. The sNG2 levels and ratio of EdU+ cell area to DAPI + cell area assessed in the *in vitro* study was analyzed using Paired T-test.

## Results

### Blood and CNS cytokine changes after systemically administered LPS

In order to confirm systemic inflammation after LPS treatment we analyzed cytokine and chemokine levels in serum 2 h and 24 h after treatment. Levels of IL-13, IL-1β, IL-4, TNF-α, IL-6 and rGro/KCwere significantly increased in rats treated with LPS compared to controls 2 hours after treatment (Table 1). After 24 hours, the IL-13, IL-4 and TNF-α levels in LPS treated rats were reduced back to baseline levels and they no longer differed significantly from controls. Although the levels of IL-1β, IL-6 and rGro/KC were still significantly elevated 24 hours after LPS treatment (Table 1), we noted a drastic decline also in theses cytokines (18- fold (p<0.001), 63- fold (p<0.001) and 150-fold (p<0.001), respectively).

**Table 1:** Serum cytokine levels (pg/ml) 2 h and 24 h after LPS and saline treatment.

	Saline2h	LPS2h	Saline24h	LPS24h
**IFN-γ**	9.65 ± 0.00^a^	19.05 ± 3.80	9.65 ± 0.00^ a^	9.65 ± 0.00^ a^
**IL-13**	17.00 ± 0.00^ a^	*44.63 ± 8.73	17.00 ± 0.00^ a^	17.00 ± 0.00^ a^
**IL-1β**	34.57 ± 7.77	***612.81 ± 101.98	26.80 ± 0.00^ a^	*33.7 ± 3.55
**IL-4**	12.3 ± 0.00^ a^	***22.63 ± 2.13	12.30 ± 0.00^ a^	19.32 ± 7.02
**IL-5**	27.50 ± 0.00^ a^	49.98 ± 9.59	27.50 ± 0.00^ a^	27.50 ± 0.00
**TNF-α**	23.63 ± 0.53	***17058.92 ± 6145.95	23.10 ± 0.00^ a^	23.10 ± 0.00^ a^
**IL-6**	35.61 ±2.53	***10920.36 ± 1522.67	35.09 ± 2.36	***76.06 ± 8.95
**rGro/KC**	145.43 ± 13.05	***19115.55 ± 684.37	135.73 ± 3.73	**306.19 ± 50.36

Values represent means ± SEM * p = 0.01, ** p = 0.001 and *** p = 0.001 indicates a significant difference compared to controls. ^a^indicate values set that were to detection limit.

To verify that the systemic inflammation led to inflammation in the CNS compartment, we moreover analyzed cytokine and chemokine levels in CSF 2 h and 24 h after LPS treatment. Analysis of CSF showed a significant increase in IL-1β, IL-4, TNF-α and IL-6, rGro/KC levels 2 hours after the LPS treatment (Table 2). In contrast to the transient cytokine elevations in blood, IL-1β and IL-6 continued to increase in CSF 24 hours after the LPS treatment (Table 2). IL-4 and rGro/KC levels, although still significantly higher compared to control, declined somewhat after 24 hours. Levels of TNF-α in LPS treated rats were no longer significantly increased compared to controls 24 hours after the LPS treatment (Table 2). Cerebrospinal fluid levels of IFN-γ, IL-13 and IL-5 were below detection limit both 2 and 24 hours after LPS treatment.

**Table 2:** CSF cytokine levels (pg/ml) 2 h and 24 h after LPS and saline treatment.

	Saline2h	LPS2h	Saline24h	LPS24h
**IFN-γ**	33.20 ± 0.00^ a^	33.20 ± 0.00^ a^	33.20 ± 0.00^ a^	33.20 ± 0.00^ a^
**IL-13**	146 ± 0.00^ a^	146 ± 0.00^ a^	146.00 ± 0.00^a^	146.00 ± 0.00^ a^
**IL-1β**	34.80 ± 0.00^ a^	*58.96 ± 10.81	34.80 ± 0.00	***294.54 ± 40.18
**IL-4**	9.86 ± 0.00^ a^	**33.81 ± 10.94	9.86 ± 0.00	**15.11 ± 1.97
**IL-5**	49.20 ± 0.00^ a^	49.20 ± 0.00	49.20 ± 0.00	49.20 ± 0.00^ a^
**TNF-α**	131.00 ± 0.00^ a^	*202.44 ± 30.59	131.00 ± 0.00^ a^	131.00 ±0.00^ a^
**IL-6**	35.20 ± 0.00^ a^	***296.90 ± 112.17	35.20 ± 0.00^ a^	***975. 23 ± 197.47
**rGro/KC**	645.00 ± 0.00^ a^	***4605.36 ± 1213.70	645.00 ± 0.00^ a^	***3250.62 ± 280.10

Values represent means ± SEM * p = 0.01, ** p = 0.001 and *** p = 0.001 indicates a significant difference compared to controls. ^a^ indicate values set that were to detection limit.

### Microglial activity in response to systemic LPS administration

**Figure 1:**
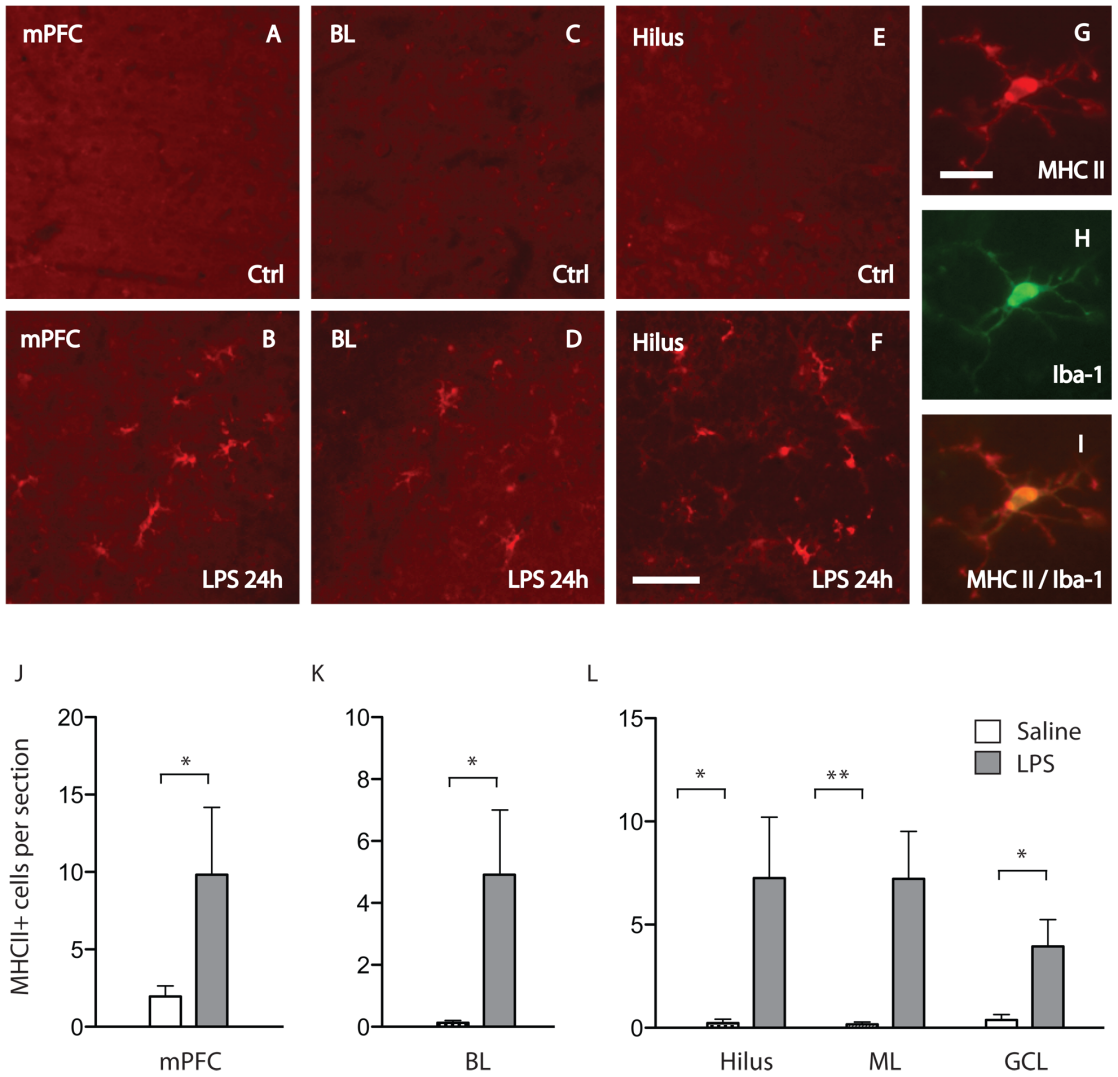
Microglial activity in response to systemic lipopolysaccharide treatment. Images in (A–L) demonstrate immunoflourescence stainings of dendritic MHCII+ cells in the medial prefrontal cortex (mPFC), basolateral nucleus of amygdala (BL) and hippocampus (Hilus) 24 hours after LPS treatment (LPS) (B, D, F) and saline treatment (Ctrl) (A, C, E). Scale bar  =  80µm. Images to the right show a microglia double-labeled with antibodies directed towards MHC II (G) and Iba-1 (H). Images (G) and (H) are superimposed in (I). Scale bar = 10 µm. Bar graphs in (J-L) show a significant increase in number of dendritic MHCII+ cells per section of mPFC (J), BL (K) and Hilus, molecular layer (ML) and granular cell layer (GCL) (L) 24 h after LPS treatment (LPS) compared to saline treatment (Saline). ** indicates a significant difference at p<0.01 level. * indicates a significant difference at p<0.05 level.

Immunohistochemical analysis of microglial activation showed that the number of dendritic MHC II expressing cells did not differ in any of the examined areas when comparing LPS treated rats with controls 2 h after treatment (Table S2). However, enhanced numbers of MHCII expressing microglia were found in all examined brain areas (i.e. BL of amygdala, ML, hilus and GCL of hippocampus and mPFC) 24 hours after the LPS treatment (figure 1).

### Alterations in the NG2 cell population after systemically administered LPS

**Figure 2:**
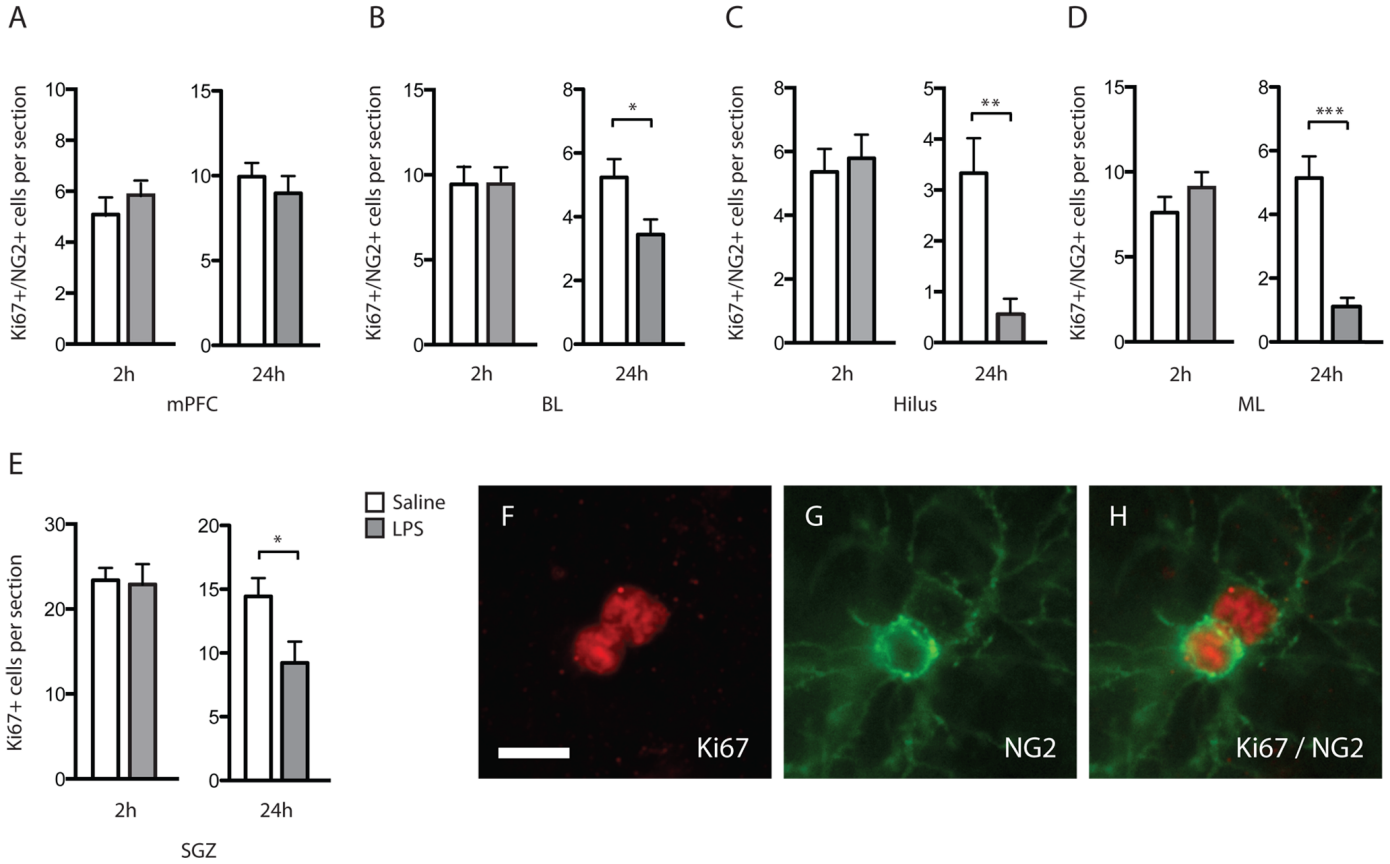
Proliferation of NG2+ cells in response to systemic lipopolysaccharide treatment. Unaltered number of NG2+/ki67+ cell numbers was found in the medial prefrontal cortex (mPFC) (A), the basolateral nucleus (BL) (B), the dentate hilus (Hilus) (C) and the molecular layer (ML) (D) 2 h after lipopolysaccharides (LPS) treatment compared to saline treatment (Saline). In contrast, a significant decreased number of NG2+/ki67+ cells were detected in the BL (B), the Hilus (C) and the ML (D), but not in the mPFC (A), 24 h after LPS treatment compared to saline treatment. The number of Ki67+ cell in the subgranular zone (SGZ) of the hippocampal granular cell layer (E) was also unaltered after 2 h, but significantly reduced 24 h after LPS compared to saline treatment. *** indicates a significant difference at p<0.001 level. ** indicates a significant difference at p<0.01 level. * indicates a significant difference at p<0.05 level. Images in (F–H) demonstrate a double immunoflourescence staining with antibodies directed against the endogenous proliferation marker Ki67 (F) and NG2 (G). Images (F) and (G) are superimposed in (H). Scale bar = 10 µm.

To evaluate the impact of systemically administered LPS on NG2 cells we investigated changes in NG2 cell proliferation and NG2 shedding. Staining against Ki67 together with NG2 (figure 2) showed no significant difference in NG2+/Ki67+ cell number in any of the examined areas when comparing LPS treated rats to controls 2 h after treatment (figure 2). However, a significant reduction in the number of NG2+/Ki67+ cells was found in BL (p = 0.028), Hilus (p = 0.002) and ML (p<0.0001) of LPS treated rats compared to controls at the later time-point at 24 h after treatment. No reduction in NG2 cell proliferation was detected in the mPFC at the 24 h survival time point (figure 2).

**Figure 3:**
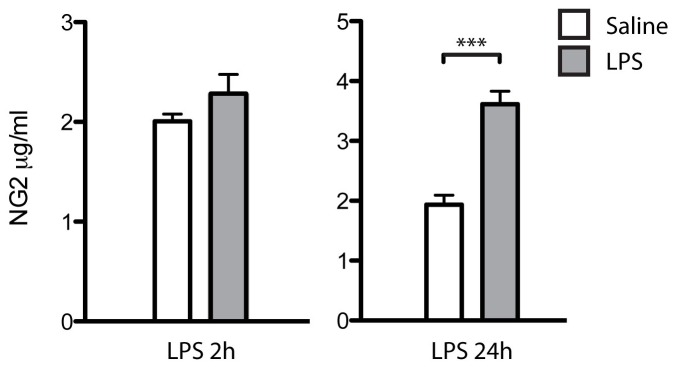
Levels of sNG2 in CSF from rats 2 h and 24 h after LPS treatment. Levels of CSF sNG2 were not significantly altered 2 h after LPS treatment compared to saline treatment (Saline), but 24 h after LPS treatment CSF sNG2 levels were significantly increased compared to saline treatment.*** indicates a significant difference at p<0.001 level.

Less than 1% of the NG2 cells displayed morphological changes in all the groups. A decline in number of Ki67+ cells in the subgranular zone (SGZ) was also detected 24 hours after LPS treatment (figure 2). Finally, no significant changes in CSF sNG2 levels were detected at 2 h, while at 24 h after treatment sNG2 levels were significantly increased in rats treated with LPS compared to controls (figure 3).

### Altered proliferation and NG2 shedding of human NG2 cells after cytokine exposure

**Figure 4:**
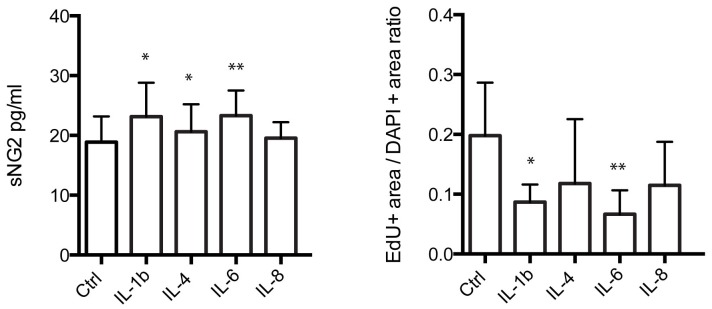
Proliferation and supernatant sNG2 levels of cultured human NG2 cells after exposure cytokines. The left bar graph shown the significantly increased sNG2 levels found in cell culture supernatant after 24 h stimulation with interleukin (IL)-1β (0.3 ng/ml), IL-6 (1.5 ng/ml) and IL-4 (150 pg/ml). The sNG2 levels were not altered in response to 20 ng/ml IL-8. The right bar graph demonstrates the significantly decreased EdU + cell area to DAPI cell area ratio after exposure to IL-1b and IL-6, but not after IL-8 or IL-4. * indicates a significant difference at p<0.05 level, ** indicates a significant difference at p<0.01 level.

Human NG2 cells were treated with IL-1β, IL-4, IL-6 and IL-8 in concentrations correlating to the CSF concentrations found at 2 hours, and left in culture for 24 hours. Analysis of the cell culture supernatant showed that IL-1β (p = 0.016), IL-4 (p = 0.038) and IL-6 p = 0.005, but not IL-8, significantly increased the levels of sNG2 (figure 4). Moreover, the proliferation analysis, where fraction of EdU positive cell area/DAPI positive cell area was used as a measure of proliferation, revealed a significant decreased proliferation of NG2 cells stimulated with IL-1β (p = 0.039), and IL-6 (p = 0.009), but not IL-4 or IL-8 (figure 4).

## Discussion

We here demonstrate significantly decreased numbers of newly formed NG2 cells in the rat amygdala and hippocampus and an almost 2-fold increase of soluble NG2 in CSF 24 h after systemic LPS treatment. At this time-point the systemic inflammation had ceased, although the CSF cytokines remained elevated which was paralleled by increased numbers of MHC II expressing microglia. Our *in vitro* experiment on human NG2 cells confirmed a direct impact of the cytokines IL-1β and IL-6 decreasing proliferation and increasing NG2 shedding.

Our results show that low-grade neuroinflammation has an inhibitory effect on NG2 cell proliferation. This result stands in contrast with previous studies demonstrating increased proliferation of NG2 cells in brain areas with severe focal brain injury [7], [8]. Nevertheless, it should be emphasized that milder neuroinflammation induced by peripherally derived cytokines does not affect proliferation of brain cells in the same way as a direct brain injury. This is exemplified by the fact that stereotactic intracranial injections with LPS increase hippocampal neurogenesis [31], whereas systemically administered LPS instead has been shown to decrease neurogenesis [32], [33], [34]. In line with these findings, also our study showed decreased number of Ki67+ cells in the SGZ (the hippocampal area were neurogenesis occurs) 24 h after LPS stimulation. Supporting our in vivo results, the culture experiment, where we stimulated human NG2 cells for 24 h with IL-4, IL-6, IL-1β or IL-8 (the latter is the human homologue for rGro/KC), further showed that the cytokines IL-6 and IL-1β specifically inhibit proliferation of NG2 cells. Both IL-1β and IL-6 are produced by activated microglia [35], [36], [37] and interestingly, an impact on NG2 cell proliferation by microglial-derived factors, but not by factors derived from astrocytes and perineuronal macrophages [38], has been observed before. Whole medium from LPS stimulated microglial cultures has previously shown to inhibit NG2 cell renewal [39]. These results suggests an intimate link between microglial activity and NG2 cell proliferation, an idea supported by the number of studies showing a simultaneously activation of NG2 cells and microglia in response to external stimuli[27], [40]. Microglia and NG2 cells are moreover commonly found spatially close to each other in the brain parenchyma [40]. Our own study demonstrating decreased NG2 cell proliferation at the same time point as the expression of MHC II is increased in microglia further support the notion of an interdependent relation between these cell types.

Microglia has been shown to express NG2 in response to systemically administered LPS [41], an event that could potentially confound our findings on NG2 cell proliferation. However, the NG2 expressing microglia cells are hypertrophic and amoebic in shape, displaying features of strongly activated microglia. In our study, we found very few NG2 cells with amoebic morphology and when counting NG2+/Ki67+ cells we only included cells that displayed the well-characterized NG2 cell ramified morphology. Hence, we found that the risk of including any potential NG2 expressing microglia in our analysis was extremely low. In addition, any confounding effects would be expected to be in the opposite direction (increased proliferation) in case microglial cells would have been included.

As mentioned in the introduction, previous studies have shown an increased amount of shedded NG2 in brain areas exposed to damage. The specific function of the shedded form of the transmembrane proteoglycan NG2 is still mainly unrevealed, but several studies indicate a role for the surface bound proteoglycan in NG2 cell proliferation. Decreased proliferation is found in the presence of antibodies directed against NG2[42], [43] and NG2 null mice pups show a delayed generation of new NG2 cells in developing white matter [44]. Moreover, renewal of NG2 cells in demyelinating lesions in the brain of NG2 null mice is decreased compared to wild type mice[45]. The proteoglycan has been suggested to affect proliferation via its ability to bind directly to the two NG2 cell mitogens, platelet derived growth factor AA (PDGF-AA) and basic fibroblast growth factor (bFGF) [46], enhancing the binding of these growth factors to their receptors[47]. It may thus be suggested that an increased shedding of NG2 leaves less NG2 on the cell surface, which in turn reduce the possibility to anchor the mitogens, consequently causing a reduced proliferation. Such a hypothesis would explain the opposing effect on NG2 cell proliferation (decreased) and sNG2 levels (increased) found in our *in vitro* and *in vivo* study. The fact that we detected increased levels of the shedded NG2 in CSF raises the possibility that it might function as a biomarker for pathological states involving NG2 cells. However it should be pointed out that NG2 cells are not the only brain cell type expressing NG2. Pericytes [48] also express NG2, something that must be kept in mind when interpreting the result of changes of sNG2 levels in CSF.

The reduced NG2 cell proliferation in response to systemic inflammation, as seen in our LPS treated rats, should be viewed from the fact that NG2 cells over the years have been shown to possess important and unique properties. Besides functioning as oligodendrocyte progenitors, they form multiple contacts with surrounding astrocytes and neurons (for review see [49]), contact nodes of Ranvier [50], stabilize neuronal synapses (for review see [51]) and promote axonal outgrowth [52], all properties dependent on the NG2 proteoglycan. Given this recognized role for NG2 cells in the neuronal network, it is reasonable to hypothesize that an inhibition of renewal of these cells and a loss of the surface bound NG2 could have an important impact on brain function and behavior. From this perspective it is interesting that downregulation of NG2 cells proliferation have been observed in rats chronically treated with corticosterone [1], [53], in rats exposed to chronic social stress [54] as well as in rats exposed to chronic mild stress [55], all treatments known to induce depressive-like behavior[54], [56]. Moreover, the down-regulation of NG2 cells proliferation in these rat models of depression were reversed by antidepressant treatments [53], [54], [55]. Also clinical studies indicate a link between depression and cells of the oligodendrocyte linage, as studies on postmortem brain tissue from depressive patients have revealed decreased number of oligodendrocytes in amygdala and prefrontal cortex [57], [58] as well as white matter changes and downregulation of oligodendrocytes genes (for review see [59]).

The reduced renewal of NG2 cells in neuroinflammation is particularly interesting given the increasing amount of clinical studies demonstrating a strong link between systemic inflammation and depressive behavior. For example, in humans certain infections induce a condition known as sickness behaviour, characterized by depressive symptoms [60]. Patients undergoing cytokine therapy often develop symptoms of depression [61] and elevated levels of cytokines (including IL-6 and IL-1β) are found in both blood and CSF from depressed patients [62], [63], [64], [65]. Finally, rats exposed to systemically administered LPS and IL-1β show depressive-like behaviour [66], [67], [68] and increased microglial activation in several depression associated brain regions (including hippocampus, amygdala and prefrontal cortex)[17], [69].

## Conclusions

Our results show that systemically administered LPS induces neuroinflammation and reduces NG2 cell proliferation in the rat brain, which can be monitored by analysis of shedded NG2 levels in CSF. These results add to a growing body of evidence that systemic inflammation might have profound effects on both the extra cellular milieu and distinct cell populations in the CNS; findings likely to be of importance for symptoms in depression and sickness behaviour.

## Supporting Information

Table S1 **Cross-sectional areas of NG2-stained brain sections from lipopolysaccharide and saline treated rats.** mPFC = medial prefrontal cortex, BL = basolateral nuclei, ML = molecular layer, Hilus = dentate hilus, GCL = granular cell layer, LPS = lipopolysaccharide. Values are presented as mean ± SEM and analyzed using student t-test. All p-value>0.05 when 2 h and 24 h groups were compared to respective saline group.
(DOCX)

Table S2 **Number of MHC II expressing microglia 2 h after saline and lipopolysaccharide treatment.** mPFC =  medial prefrontal cortex, BL = basolateral nuclei, ML = molecular layer, Hilus = dentate hilus, GCL = granular cell layer, LPS = lipopolysaccharide. Values are presented as mean ± SEM and analyzed using student t-test. All p-value>0.05 when 2 h and 24 h groups were compared to respective saline group.
(DOCX)
