# Supplementary material for: Pro-Inflammatory Cytokines Reduce the Proliferation of NG2 Cells and Increase Shedding of NG2 In Vivo and In Vitro
Source: PLoS One. 2014 Oct 6;9(10):e109387. doi: 10.1371/journal.pone.0109387 (PMC4186831; doi:10.1371/journal.pone.0109387)
Supplement: Table S1 — Cross-sectional areas of NG2-stained brain sections from lipopolysaccharide and saline treated rats. mPFC = medial prefrontal cortex, BL = basolateral nuclei, ML = molecular layer, Hilus = dentate hilus, GCL = granular cell layer, LPS = lipopolysaccharide. Values are presented as mean ± SEM and analyzed using student t-test. All p-value>0.05 when 2 h and 24 h groups were compared to respective saline group. (DOCX) [file pone.0109387.s001.docx]

|  | **Saline 2h** | **LPS 2h** | **Saline 24h** | **LPS 24h** |
| --- | --- | --- | --- | --- |
| **mPFC (mm^2^)** | 3.66 ± 0.10 | 3.71 ± 0.09 | 3.14 ± 0.12 | 3.19 ± 0.19 |
| **BL (mm^2^)** | 1.01 ± 0.14 | 1.02 ± 0.14 | 0.94 ± 0.34 | 0.89 ± 0.27 |
| **ML (mm^2^)** | 0.59 ± 0.19 | 0.57 ± 0.11 | 0.82 ± 0.19 | 0.80 ± 0.16 |
| **Hilus (mm^2^)** | 0.39 ± 0.13 | 0.38 ± 0.08 | 0.43 ± 0.17 | 0.43 ± 0.08 |
| **GCL (mm^2^)** | 0.19 ± 0.07 | 0.21 ± 0.14 | 0.15 ± 0.10 | 0.15 ± 0.06 |

**Table S1.** Cross-sectional areas of NG2-stained brain sections from lipopolysaccharide and saline treated rats
