# Supplementary material for: Pro-Inflammatory Cytokines Reduce the Proliferation of NG2 Cells and Increase Shedding of NG2 In Vivo and In Vitro
Source: PLoS One. 2014 Oct 6;9(10):e109387. doi: 10.1371/journal.pone.0109387 (PMC4186831; doi:10.1371/journal.pone.0109387)
Supplement: Table S2 — Number of MHC II expressing microglia 2 h after saline and lipopolysaccharide treatment. mPFC = medial prefrontal cortex, BL = basolateral nuclei, ML = molecular layer, Hilus = dentate hilus, GCL = granular cell layer, LPS = lipopolysaccharide. Values are presented as mean ± SEM and analyzed using student t-test. All p-value>0.05 when 2 h and 24 h groups were compared to respective saline group. (DOCX) [file pone.0109387.s002.docx]

**Table S2.** Number of MHC II expressing microglia 2 h after saline and lipopolysaccharide treatment.

|  | **mPFC (no)** | **BL (no)** | **ML (no)** | **Hilus (no)** | **GCL (no)** |
| --- | --- | --- | --- | --- | --- |
| **Saline** | 0.19 ± 0.08 | 0.08 ± 0.05 | 0.28 ± 0.10 | 0.16 ± 0.07 | 0.83 ± 0.30 |
| **LPS** | 0.42 ± 0.20 | 0.06 ± 0.04 | 0.22 ± 0.12 | 0.36 ± 0.19 | 1.12 ± 0.63 |
